# Drug resistance and treatment failure in leishmaniasis: A 21st century challenge

**DOI:** 10.1371/journal.pntd.0006052

**Published:** 2017-12-14

**Authors:** Alicia Ponte-Sucre, Francisco Gamarro, Jean-Claude Dujardin, Michael P. Barrett, Rogelio López-Vélez, Raquel García-Hernández, Andrew W. Pountain, Roy Mwenechanya, Barbara Papadopoulou

**Affiliations:** 1 Department of Physiological Sciences, Laboratory of Molecular Physiology, Institute of Experimental Medicine, Luis Razetti School of Medicine, Universidad Central de Venezuela, Caracas, Venezuela; 2 Department of Biochemistry and Molecular Pharmacology, Instituto de Parasitología y Biomedicina López-Neyra, Spanish National Research Council (IPBLN-CSIC), Granada, Spain; 3 Molecular Parasitology Unit, Department of Biomedical Sciences, Institute of Tropical Medicine, Antwerp, Belgium; 4 Wellcome Centre for Molecular Parasitology, Institute of Infection, Immunity and Inflammation, University of Glasgow, Glasgow, United Kingdom; 5 Department of Infectious Diseases, National Referral Unit for Tropical Diseases, Ramón y Cajal University Hospital, Madrid, Spain; 6 Department of Biomedical Sciences, School of Veterinary Medicine, University of Zambia, Lusaka, Zambia; 7 Research Center in Infectious Diseases, CHU de Quebec Research Center and Department of Microbiology-Infectious Disease and Immunology, University Laval, Quebec, Canada; University of Antwerp, BELGIUM

## Abstract

Reevaluation of treatment guidelines for Old and New World leishmaniasis is urgently needed on a global basis because treatment failure is an increasing problem. Drug resistance is a fundamental determinant of treatment failure, although other factors also contribute to this phenomenon, including the global HIV/AIDS epidemic with its accompanying impact on the immune system. Pentavalent antimonials have been used successfully worldwide for the treatment of leishmaniasis since the first half of the 20th century, but the last 10 to 20 years have witnessed an increase in clinical resistance, e.g., in North Bihar in India. In this review, we discuss the meaning of “resistance” related to leishmaniasis and discuss its molecular epidemiology, particularly for *Leishmania donovani* that causes visceral leishmaniasis. We also discuss how resistance can affect drug combination therapies. Molecular mechanisms known to contribute to resistance to antimonials, amphotericin B, and miltefosine are also outlined.

## Author summary

Chemotherapy is central to the control and management of leishmaniasis. Antimonials remain the primary drugs against different forms of leishmaniasis in several regions. However, resistance to antimony has necessitated the use of alternative medications, especially in the Indian subcontinent (ISC). Compounds, notably the orally available miltefosine (MIL), parenteral paromomycin, and amphotericin B (AmB), are increasingly used to treat leishmaniasis. Although treatment failure (TF) has been observed in patients treated with most anti-leishmanials, its frequency of appearance may be important in patients treated with MIL, which has replaced antimonials within the kala-azar elimination program in the ISC. AmB is highly efficacious, and the associated toxic effects—when administered in its free deoxycholate form—are somewhat ameliorated in its liposomal formulation. Regrettably, laboratory experimentation has demonstrated a risk of resistance towards AmB as well. The rise of drug resistance impacts treatment outcome, and understanding its causes, spread, and impact will help us manage the risks it imposes. Here, we review the problem of TF in leishmaniasis and the contribution of drug resistance to the problem. Molecular mechanisms causing resistance to anti-leishmanials are discussed along with the appropriate use of additional available drugs, as well as the urgent need to consolidate strategies to monitor drug efficacy, epidemiological surveillance, and local policies. Coordination of these activities in national and international programs against leishmaniasis might represent a successful guide to further research and prevention activities.

## Introduction

The leishmaniases are complex diseases of (sub)tropical regions of the world caused by *Leishmania* spp. (Protozoa, Kinetoplastida, Trypanosomatidae) and spread by sand flies. The World Health Organization (WHO) considers the leishmaniases to be prominent among the global causes of death by infectious diseases [1].

Clinical manifestations produced by *Leishmania* comprise the visceral (VL) and tegumentary forms. The tegumentary forms of the disease include the cutaneous (CL), diffuse (DCL), and mucocutaneous (MCL) leishmaniases [1], but infections remain asymptomatic in many cases [2, 3]. *Leishmania* may also appear as an opportunistic parasite in immunosuppressed individuals. Chemotherapy constitutes the main approach to manage the disease although it is generally not applied to asymptomatic subjects. For a number of other neglected tropical diseases, mass drug administration—even without diagnosis—is possible, given the safety of particular drugs used in these circumstances (e.g., praziquantel in schistosomiasis and ivermectin for lymphatic filariasis). The combined problems of parenteral administration and toxicity of anti-leishmanials precludes such programs for leishmaniasis. Furthermore, the selection of resistant parasites carrying genetic mutations that lessen the parasite's response to drugs may emerge upon mass drug administration.

*Leishmania* has an intricate life cycle, and one of the developmental forms, the amastigote, dwells within immunological cells of the mammalian host, which adds to the challenge of accessing the parasites with specific drugs. Nevertheless, the aim of chemotherapy is to kill intracellular parasites; therefore, chemotherapy remains the best means available to cure the disease [4].

Antimonials (sodium stibogluconate [SSG]) are the primary drugs employed against leishmaniasis. They have been in use since the 1920s. These toxic compounds have a narrow therapeutic window, and their use has been largely superseded in the ISC, where resistance has become widespread. However, they are still in use in other regions of the world, including Latin America and East Africa [5,6].

MIL has replaced SSG in the ISC in the context of the kala-azar elimination program, but efficacy of this drug as well had already dropped within a decade of its introduction [7, 8]. Initially, this decrease in MIL efficacy could not be related to increasing parasite resistance to the drug [8], although recently a few resistant clinical isolates have been described, first in France in an HIV-coinfected patient and later in two Indian patients [9, 10, 11].

AmB is highly efficacious but relatively toxic when injected in its free deoxycholate form. Administration in a liposomal formulation ameliorates the toxicity risk, although the high cost of this formulation has left its range of use restricted; this is the case even though it is provided free of charge to WHO for use in strategically important areas by its manufacturers (Gilead Sciences; with up to 350,000 vials over the next five years). Unfortunately, a risk of resistance is becoming apparent for AmB as well (discussed below).

Paromomycin has a relatively restricted range of targeting *Leishmania* species, and the situation regarding resistance in the field is unclear, although laboratory-derived resistant isolates have been created [12–15].

Even combination therapies are not immune from selection of resistance, which poses potential challenges to the WHO’s proposed next generation of combination therapies in the treatment of leishmaniasis.

It is critical to consider that TF and drug resistance (DR) are not necessarily synonymous. TF goes far beyond DR, with numerous factors in the host (such as immunity or nutritional status), the parasite (e.g., whether or not the parasite resides in tissues not accessible to drugs), and the environment (e.g., global warming contributing to the expansion of the disease to new geographical areas) influencing treatment outcomes. Notwithstanding, DR is a fundamental determinant of treatment outcome, and understanding the mechanisms whereby parasites become resistant to drugs is essential. In this Review, we discuss the phenomenon of DR associated with SSG, AmB, and MIL and outline the molecular mechanisms associated with the selection of resistance in combination therapies, as well as the possible consequences of emergent resistance on the use of these drugs in the field.

## TF beyond DR

A growing number of questions apply to treatment options for leishmaniasis. For example, why do patients with the same clinical form of the disease, living in the same areas, infected by the same species of *Leishmania*, and treated with the same drug regimen differ in their response to treatment? What are the causes of TF? To help address these questions from the parasitological point of view, two key requirements are (1) to find a sensitive and practical technique to determine the persistence, or the elimination, of viable parasites in clinical samples after treatment and (2) to define and standardize tests of sensitivity and resistance in vitro.

**Fig 1:**
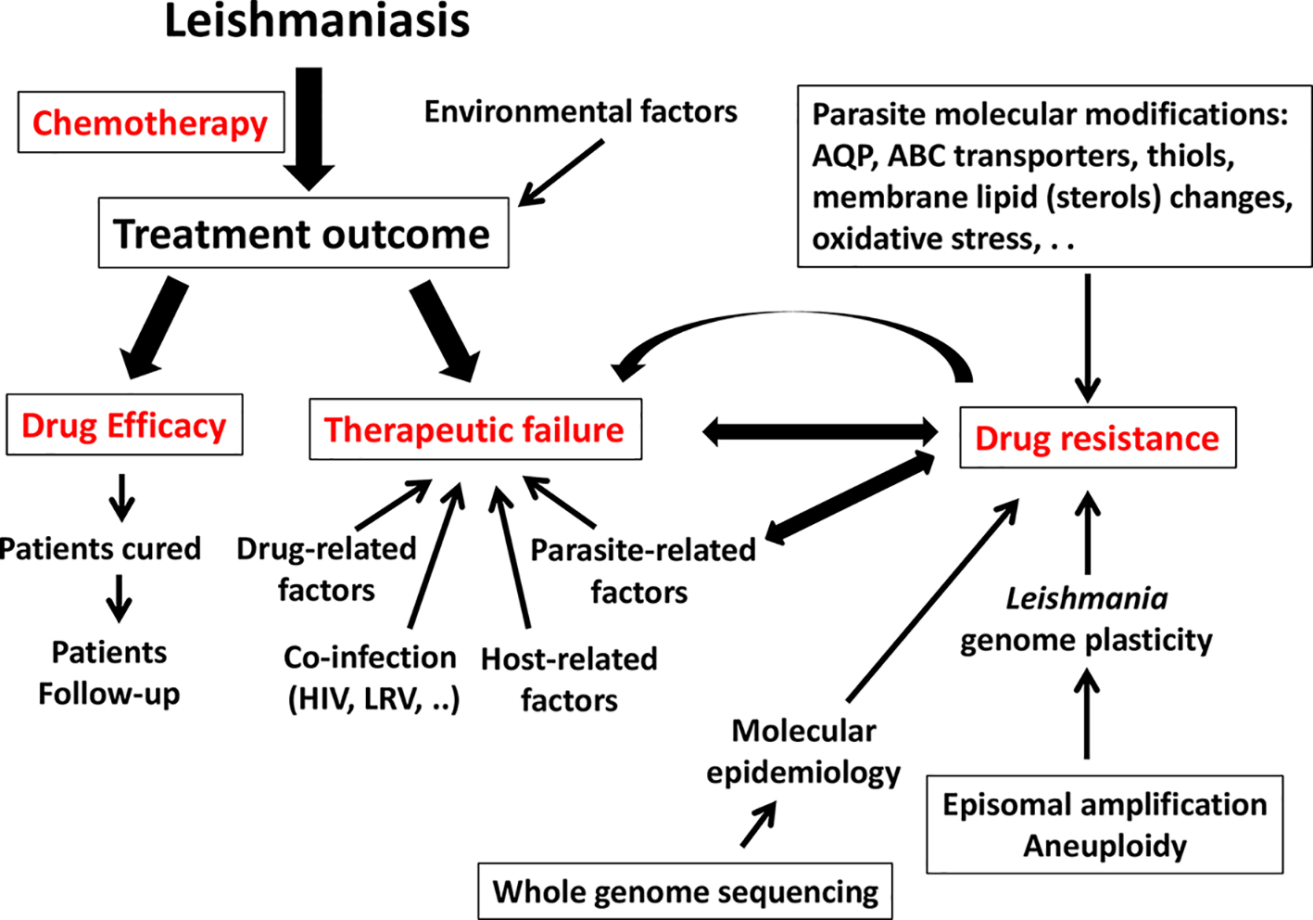
Summary of the factors that influence TF and DR in *Leishmania*. ABC, ATP-binding cassette; AQP, aquaporin; DR, drug resistance; LRV, *Leishmania* RNA virus; TF, treatment failure.

Numerous features are known to impact the final therapeutic outcome (see Fig 1).

Host factors play a role. For example, because an effective immune response is required to support anti-leishmanial drugs, patients with immunodeficiency can be particularly hard to cure [16]. Less extreme natural variation in host immunological response can also influence the ability of a drug to work. To date, little has been done to assess the effect of individual variation in pharmacokinetics or other drug-related responses in the host. Differences between individuals are normal for most drugs, and stratified responses are clearly observed for the leishmaniases. Examples include when comparing children versus adults or males versus females [17, 18, 19] and in instances in which a patient who may have failed initial treatment responds to a second cycle of a drug given at a higher dose [20].

It is also clear that parasite factors other than explicitly acquired resistance can also play a role. These factors comprise the inherent virulence of the infecting isolate of *Leishmania* [21] and—in some instances—parasite infection by RNA viruses [22, 23], whose occurrence can stimulate a different kind of immunological response in the host [24]. TF may occur with susceptible strains in immunocompromised patients [25, 26], and ill-defined regional differences in response are known [27, 28]. Finally, TF may occur in similar clinical cases that respond to a given therapy in some patients but fail in others when the causative *Leishmania* species or strain is different [29].

Factors related to the drug itself can be crucial as well. Inappropriate dosing by inexperienced health workers (or self-administration in the case of MIL) may lead to subtherapeutic dosage and introduce the risk of selecting parasites resistant to those drugs [18]. Administering agents in difficult-to-work-in environments can also be a problem, e.g., with the application of a product that is faulty or the expiration of its activity due to long-term storage in inappropriately hot conditions [30, 31].

"To cure or not to cure" is the main issue in clinical practice. The patient must be evaluated at the end of treatment to determine the outcome. Should this evaluation be solely clinical, or should it also include parasitological confirmation? While clinical evaluation is mandatory, and some clinical scores have been proposed for CL [32], parasitic evaluation (test of cure [TOC]) includes aggressive, sophisticated, and costly tests (bone marrow biopsy, splenic aspirate, parasite culture, and PCR techniques, among others). It is, nevertheless, generally required, depending on the specific circumstances surrounding the patient. New, confirmatory assays are emerging that appear promising for the prognosis of treatment outcome in VL. These include rapid diagnostic tests capable of detecting anti-*Leishmania* IgG1 [33] and loop-mediated isothermal amplification (LAMP) [34]. Unfortunately, molecular diagnosis remains a challenge in American tegumentary leishmaniasis because the variety of causative *Leishmania* species may confuse the outcome. This is especially true when parasites are scarce in tissues, as is the case for *L (V*.*) braziliensis* in CL and MCL patients.

If inflammatory signs are absent, and complete scarring of the lesion (determined by the size of the inner and outer borders of the wound) or re-epithelialization of ulcerative lesions at three-month follow-up occurs, then definitive cure for CL can be claimed. For VL, a good clinical response correlates to the normalization of temperature; disappearance of symptoms; decreased size of the liver and spleen; increased counts in peripheral blood of leukocytes, hemoglobin, and platelets; and increased appetite and weight gain [20].

Parasitological cure is defined by the absence of *Leishmania* parasites detected by microscopy or culture from tissue samples (skin, spleen, bone marrow, or lymph nodes). The detection of parasite DNA by PCR in tissues is substantially more sensitive than conventional parasitological techniques, but it may give false positive results when performed too early because of the persistence of nonviable *Leishmania* parasites. Nevertheless, RNA targets have been exploited for the assessment of *Leishmania* viability, e.g., by reverse transcription PCR (RT-PCR) amplification of the 7SL RNA transcript, which proved a rapid and efficient method to detect and quantify viable parasites in tissue samples [35].

Quantitative PCR in peripheral blood can also be useful for measuring the initial parasitic load and for monitoring VL responses to treatment (but these tests are not standardized and are not widely available for clinical use). Biomarkers—including cytokines, their receptors, and acute-phase proteins, among others—are under evaluation. Serological tests are generally not useful for follow-up, given the persistence of seroconversion post cure [36].

In drug trials, TOC is usually performed one month after the last dose of treatment, which may be too early because residual parasites can be found in patients who, when diagnosed, had a very high parasite load (this may be the case of immunosuppressed patients). In clinical practice, TOC is generally not recommended in patients showing a timely clinical response.

Two main forms of TF can be defined as follows: (1) nonresponse or the persistence of symptoms at the end of treatment and (2) relapse, that is, a second episode after initial apparent patient cure. In both cases, clinical symptoms might be verified by parasitological means. Because relapses usually occur within the first 6 to 12 months after the end of treatment, following the patients for at least one year before considering them as cured would seem warranted [8].

In conclusion, the relative importance of parasite resistance, as measured using currently available techniques, is still unclear in clinical practice because host immunological or clinical features are at least as likely to underpin TF. Assays to determine whether parasites are sensitive or resistant to drugs have not been standardized and are not available in the vast majority of medical clinics and/or centers where the disease is treated. Theoretically, these assays should be essential in monitoring drug sensitivity and/or resistance in specific geographical areas, but they are of little use in clinical practice and thus only rarely requested in particular relapsing cases. In fact, experimental assays that might answer this key question can currently only be used in an epidemiological context, for surveillance, not for diagnosis. Substantial improvements in techniques for molecular diagnostics are needed before the implementation of such tests into clinical practice is feasible.

Last but not least, information on drug sensitivity is essential when designing therapeutic guidelines in a given geographical area. Thus, although TF certainly goes far beyond DR, treatment outcome should be considered in the context of DR as an important contributing factor.

## What do we mean by the term “drug resistance”?

As mentioned previously, SSG were for a long time the mainstay treatment in the fight against leishmaniasis but were abandoned in 2005 in some areas, including the ISC, due to TF related to DR. SSG remain, nevertheless, the drugs of choice in many countries around the world [4]. MIL, however, is an alternative drug that was developed in the mid-1980s and first became available in 2002 for VL in India, then in 2005 for CL in Latin America [37]. Recent reports indicate that the effectiveness of MIL in India and Nepal is decreasing [7, 8]. In these areas, the drug has now been replaced by liposomal AmB for the kala-azar elimination program [6].

Classically, resistance emerges as genetic mutations that lessen the parasite’s response to a drug when the parasite is under drug pressure. This situation is easy to translate to selective conditions in the field. However, an intriguing point to consider is the description of *Leishmania* parasites resistant to SSG, even in cases in which parasites have not been exposed to the drug [38]. Antimony is a heavy metal whose action against *Leishmania* shares characteristics with the related heavy metal arsenic. Under experimental conditions, resistance selected to arsenic renders parasites cross-resistant to antimony. In North Eastern India, high levels of arsenic in drinking water may have led to selection of parasites with reduced sensitivity to both heavy metals, a plausible explanation for the spread of antimony resistance in this region [39]. This has been evaluated in a retrospective epidemiological survey performed in Bihar, India, the conclusions of which suggest that arsenic-contaminated groundwater may well be associated with SSG TF. The failure rate with SSG was found to be 59%. Of note, patients living in areas with high mean local arsenic levels showed a higher risk (albeit statistically nonsignificant) to SSG TF than patients living in areas in which the wells had low arsenic concentrations <10 μg/L [40].

## Molecular mechanisms of DR to antimonials

**Fig 2:**
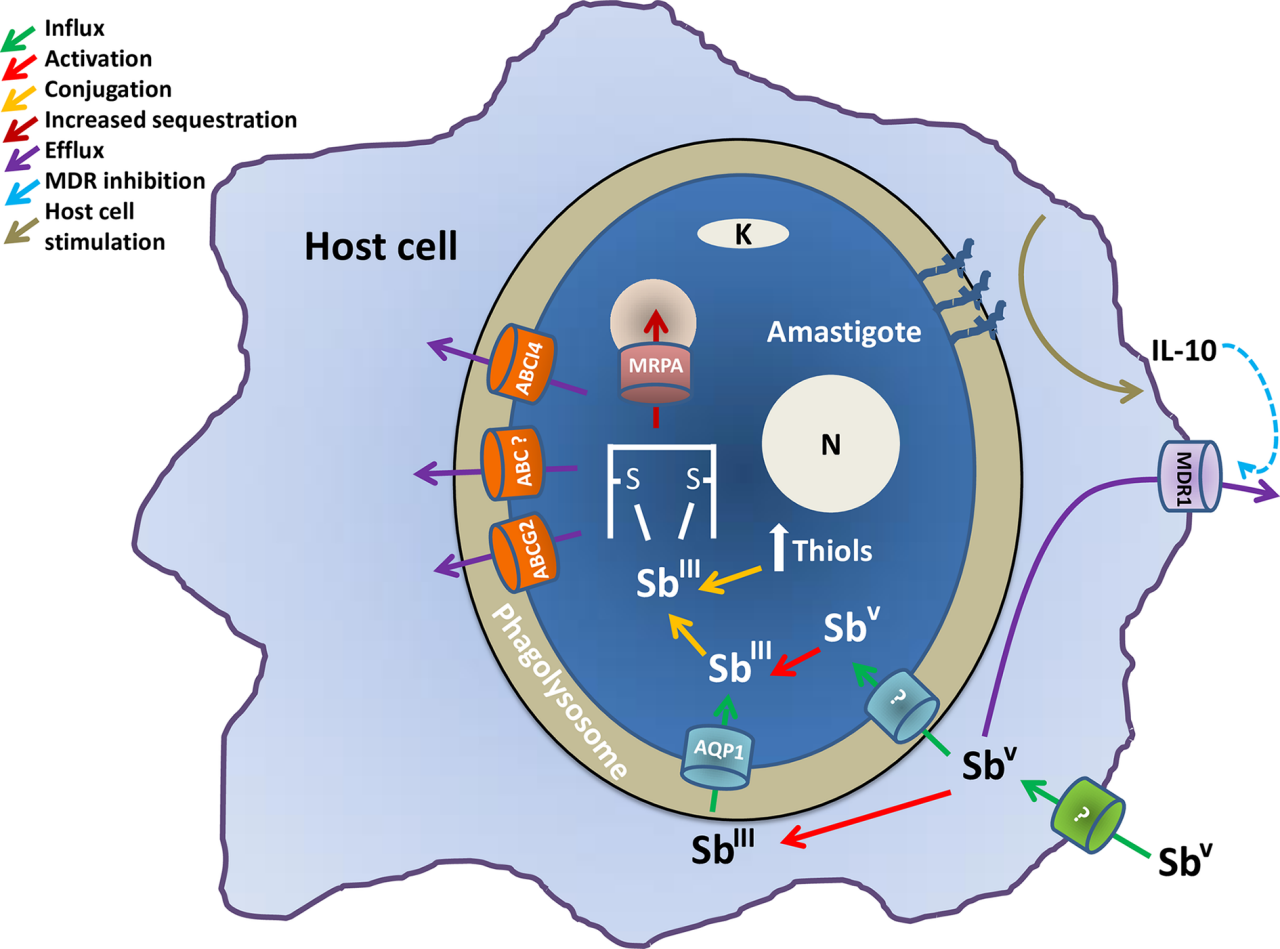
Molecular mechanisms of antimonial DR in *Leishmania*. The figure illustrates an amastigote inside a phagolysosome of the macrophage host cell. It explains the activation and influx methods used by antimonials to enter the parasite and the intracellular mechanisms used by the parasite to express the resistant phenotype. ABC, ATP-binding cassette; AQP, aquaporin; DR, drug resistance; IL-10, interleukin 10; MDR1, multidrug resistance protein 1.

A significant amount of work has been devoted to understanding how SSG exert their selective action against *Leishmania* and how resistance emerges (see Fig 2). To obtain the anti-leishmanial products SSG and potassium antimony tartrate, a chemical reaction must occur between various components; e.g., stibonic and gluconic acids are combined to produce SSG, i.e., it is a complex chemical mixture and not a single compound [57]. Pentavalent antimony (Sb^V^) must be reduced to its trivalent form (Sb^III^) for activity. Some of this reduction occurs within the host macrophage, and the resultant Sb^III^ enters via the AQP1 membrane carrier. Sb^V^ also enters the parasite via another, as yet uncharacterized, carrier mechanism and is further reduced to Sb^III^ within the cell.

Antimony accumulation is lower in resistant parasites when compared to sensitive parasites, although it is unclear whether or not actual levels of accumulation relate to sensitivity in wild-type cells [41]. Overexpression of AQP1 renders the parasites hypersensitive to Sb^III^, whereas gene deletion renders them resistant [42], and reduced levels of AQP1 expression also relate to resistance [43]. As described later, a mutation to AQP1 that renders the gene inactive is associated with a high level of antimony resistance in India.

Diminished biological reduction of Sb^V^ to Sb^III^, decreased internalization of the drug, and increased levels of trypanothione, which provides increased thiol redox potential, have also been implied in resistance [44–46]. Overexpression of ATP-binding cassette (ABC) transporters involved in ATP-dependent transport of a variety of molecules across biological membranes has also been shown to influence the efflux of drugs and can play a role in antimony resistance in *Leishmania*. MRPA is an ABC transporter localized in membrane vesicles close to the flagellar pocket [47, 48]. Its overexpression confers antimonial resistance both in amastigotes and promastigotes by sequestering thiol–metal conjugates in intracellular vesicles.

The molecular epidemiological analysis of field isolates in the ISC also showed that an intrachromosomal amplification of MRPA that probably emerged in the mid-19th century predisposed *L*. *donovani* to develop resistance to SSG (see below).

Trypanothione is an unusual bis-glutathionyl spermidine adduct found uniquely in the phylogenetic group that includes *Leishmania*. Trypanothione binds to Sb^III^, and the resultant metal–trypanothione conjugates are either sequestered into an intracellular organelle by MRPA or extruded from the cell by other efflux pumps [49]. Overexpression of key enzymes in trypanothione synthesis (ornithine decarboxylase and gamma-glutamylcysteine synthase) has also been associated with resistance in conjunction with overexpression of MRPA [49].

PRP1 is another ABC transporter protein originally identified due to an ability to confer resistance to pentamidine [50]. It has been postulated that it can also confer resistance to antimony, although its localization and mechanism of resistance have not yet been determined [48]. Other ABC transporters in *Leishmania*, including ABCI4 [51] and ABCG2 [52], can also contribute to antimony resistance by the efflux of the drug as conjugated metal–thiol conjugates.

Finally, overexpression of tryparedoxin peroxidase has been associated with Sb^III^ resistance [53–55] through elevated levels of reduced intracellular thiols. Many resistant mutants exhibit significantly increased levels of intracellular thiols, including cysteine, glutathione, and trypanothione, and lowering intracellular thiol levels in SSG-resistant (SSG-R) mutants can cause partial reversion of the resistance phenotype. Antimony induces oxidative stresses within the cells; therefore, an increased ability to deal with such stresses contributes to resistance. It is, then, clear that a variety of different mechanisms can all contribute—to varying degrees—to the ability of *Leishmania* parasites to resist antimony in a complex fashion.

Also of note is the fact that some changes to parasite physiology can also influence resistance by orchestrating changes to macrophage biochemistry, including increases in the macrophage’s ability to extrude Sb^V^- SSG [56]. Complex glycans present on the surface of SSG-R parasites but not on SSG-sensitive parasites contribute through a cascade of cellular events to the up-regulation of the anti-inflammatory cytokine interleukin 10 (IL-10) levels in the host. This, in turn, provokes overexpression of multidrug resistance protein 1 (MDR1) efflux systems in the macrophage [56], which diminishes the total levels of antimony reaching the parasites. This also shows how coinfection status could influence treatment response; e.g., other agents that stimulate IL-10 levels could diminish the response of *Leishmania* to SSG.

The complexity of host and parasite factors that affect outcomes of *Leishmania* infections treated with SSG is becoming clear and, as outlined above, understanding of mechanisms that have been observed in laboratory analyses have been shown to be pertinent in explaining the emergence and spread of SSG resistance in the field.

## Molecular epidemiology of DR in *L*. *donovani*

The main factors behind the epidemiology of the leishmaniases as (re)emerging diseases spreading to new geographical areas include human-made and environmental changes, changes in host immune status, and TF (due to either DR or other factors). These factors may act synergistically to enhance the endemicity of the disease. For example, immunocompetence of patients impacts the emergence of DR [9]. Demographic changes, with movement of infected people from endemic rural regions to sprawling conurbations, lead to increasing incidence of HIV–*Leishmania* coinfection, as is the case in Brazil [58]. The epidemiological and clinical context of any given endemic region can thus influence DR and should be considered in depth. Resistance to SSG, in *L*. *donovani* of the ISC, is the best-documented example of molecular epidemiology of DR in leishmaniasis and is discussed below.

Poor socioeconomic conditions [59] seem to be a fundamental contributory factor for antimonial resistance in the North Bihar region of India. Toxicity of SSG and suboptimal dosing (<10 mg/kg/day), including a steady-step but smooth increase and even recommendations of drug-free intervals, may also have contributed to this situation by presenting conditions amenable to the selection of resistance. Parasites with varying susceptibility to SSG coexist in the field and also influence the variable responses that occur in different geographical areas [60].

Studying the molecular epidemiology of VL in the ISC has been hindered by the lack of high-resolution tools enabling analysis of genetic markers associated with resistance. DNA microsatellite sequences, which are generally useful for *Leishmania* genotyping, could not discriminate most of the strains in the ISC [61] because of the high genetic homogeneity of the ISC population. However, the advent of whole genome sequencing (WGS) provided the level of resolution needed to answer questions regarding the molecular epidemiology of *L*. *donovani* in the ISC, both generally and in the context of DR.

This was demonstrated in 2016 by Imamura et al. [62] in a pioneering WGS study of 204 Indian and Nepalese isolates that had been well documented both clinically and epidemiologically. The fundamental results of this study were as follows:

1. The sampling revealed the existence of a core population (about 93% of all isolates) endemic in the lowlands and a small, genetically distinct population (called ISC1 or “Yeti”) encountered in hilly districts of Nepal.
2. The core population appears to have a recent history—the molecular data suggest an origin in around 1850 followed by a series of bottlenecks and expansions, resulting in nine subpopulations (named ISC2 to ISC10); six of them were identified as congruent monophyletic groups, and three demonstrated signs of genetic recombination.
3. The last expansion within the core population dates from the 1960s, which coincided with the aftermath of a regional DDT-spraying campaign. It was during this expansion that SSG began its gradual decrease in efficacy, linked to the emergence and spread of SSG resistance.
4. SSG-R strains are present in different subpopulations, indicating the independent emergence of DR on several occasions; in ISC5, the emergence of DR was followed by a clonal spread, and it is within ISC5 that most SSG-R strains (associated with TF) were encountered. Interestingly, strains with intermediate SSG-R were described in the “recombinant” groups, indicating that genetic recombination contributes to the spreading of resistance genes.
5. Two molecular adaptations seem to be particularly relevant in this study. First, the 191 strains belonging to the core population have an intrachromosomal amplification of the “H-locus,” containing an ABC transporter termed MRPA, a known player in SSG resistance (see section above).

The occurrence of this amplicon in the whole core population indicates that it is an ancestral character and was already present in 1850; its occurrence also suggests that it constitutes a preadaptation that facilitated the emergence of true resistance once SSG was introduced many years later. How it was selected at a time when SSG was not yet in use remains to be elucidated, although the relationship between arsenic in drinking waters and selection of Sb^III^ resistance discussed above demonstrates how environmental stimuli can influence DR in leishmaniasis.

A second level of adaptation occurred specifically in ISC5, where all of the sequenced isolates contain a homozygous two–base-pair insertion in the gene encoding *AQP1* (another known player in antimony resistance, see above) [42]. AQP1 activity is abolished in the resistant lines, and this is probably responsible for the high level of resistance observed. Interestingly, hybrid strains showing intermediate SSG-R were heterozygous for the *AQP1* insertion [42].

WGS offers unprecedented insight into the molecular epidemiology of *Leishmania*, in particular in the context of DR. As such, this method should be implemented in surveillance schemes, when and where it is available. Of course, molecular tracking could be simplified and new assays translated from this genomic knowledge, as seen with the single locus genotyping (SLG) for the detection of ISC groups [63] or a PCR assay for the detection of the 2-nt insertion in AQP1. However, such targeted assays do have limitations. For example, when SLG was evaluated in Nepal, about 50% of the isolates could not be typed, possibly because other genotypes circulating in this region are not detected by SLG [63].

Furthermore, different molecular modifications can alter the functionality of a gene such as *AQP1*. For example, a reduction in number of the chromosome bearing the gene, deletion of the gene, or different single nucleotide polymorphisms (SNPs) affecting key residues can all affect gene function, a concept recently introduced under the name of “Many Roads to Drug Resistance” [64]. A PCR test specifically targeting the AQP1 2-nt insertion will not detect the other genomic alterations and thus would fail to identify other types of mutation affecting this gene.

Therefore, untargeted methods such as WGS remain the best choice until a full catalogue of all mutations that can cause resistance is created. Recent development and validation of WGS protocols allowing direct sequencing in clinical samples represent a promising and currently needed alternative because they avoid the problems and biases related to isolation and cultivation of parasites—and new markers in parasites found in situ may emerge.

Finally, transmission patterns of different *Leishmania* species can impact the spread of resistance. This is clearly exemplified by the role of reservoirs that are generally untreated, such as asymptomatics or post–kala-azar dermal leishmaniasis (PKDL) patients for *L*. *donovani* and animal reservoirs both for *L*. *braziliensis* and *L*. *tropica*. Transmission into conditions where selective pressure is removed can slow down the rate at which resistance is fixed within the population [65–67].

Lessons from WGS to understand the epidemiology of DR to SSG in the ISC should influence how information is gathered on the increase and spread of resistance to other drugs as well and underpin the development of surveillance strategies for implementation into leishmaniasis control programs globally.

## *Leishmania* genome plasticity and DR

*Leishmania* has a highly plastic genome (see Fig 1), with an enormous potential for aneuploidy, local copy number variations (CNVs) of specific loci, and extrachromosomal circular or linear amplification of sets of genes [68, 69]. This plasticity relates to the *Leishmania* genome containing numerous pairs of short repeats flanking groups of genes, which promote genome-wide stochastic adaptive DNA amplification [70]. Such variability may play key roles in the adaptive and evolutionary biology of the parasite. For example, it permits an increase in the quantity of transcripts of given genes, a good solution for an organism that cannot regulate transcriptional initiation [71]. Additionally, this plasticity allows—at low risk—the creation of genetic diversity among the additional copies of amplified genes, a useful adaptive strategy for immunogens like GP63 [72]. In this context, it is not surprising that genome plasticity has also been exploited by the parasite to generate DR.

In experimental conditions, episomal amplification of different sets of genes is a molecular adaptation often found in parasites selected for DR [73]. This is the case, for example, for MRPA in the context of SSG resistance [74, 75] and in methotrexate-resistant parasites of dihydrofolate reductase-thymidylate synthase [76, 77]; in both cases, increased activity of the proteins encoded by these genes leads to resistance. By contrast, episomal amplification has not been observed for AQP1, which is because the AQP1 transporter imports drug; therefore, loss of activity is required for resistance associated with this gene. The same is true for the MIL-resistant *Leishmania donovani* transporter (LdMT1) involved in MIL uptake [78].

Experimental episomal amplification can emerge rapidly, but it also generally disappears rapidly, when the drug pressure is stopped. In natural conditions, gene amplification is also observed, e.g., the H-locus containing the MRPA gene [79] and a mitogen-activated protein kinase 1 (MAPK1) gene in the core population of *L*. *donovani* [9] on the ICS. In this case, however, it is an intrachromosomal amplification rather than episomal amplification, which is likely explained by the higher stability offered by amplification within a chromosome.

A second strategy exploited by *Leishmania* to alter copy number of particular genes is to increase copy number of the entire chromosome upon which they reside, generating aneuploidy. Under laboratory conditions, the emergence of aneuploidy usually occurs early under drug selection, while other mutations—including SNPs, indels, or gene deletions—arise later. Aneuploidy allows increasing or decreasing gene dosage, according to the driver gene(s) need of gain- or loss-of-function. For example, for LdMT (one of the main transporters accountable for MIL uptake in *L*. *donovani*—for which DR is linked to loss-of-function through diminished drug uptake)—a decrease of somy is selected in some resistant parasites [78]. In the case of MRPA (DR linked to gain-of-function through drug sequestration), an increase in chromosome copy number is observed [75].

Of noteworthy importance is that the extent of aneuploidy is not the same among individual cells constituting a strain. This mosaicism allows the population to have cells in different genomic states, the fittest being selected depending on the selective conditions [80]. Extensive aneuploidy is also observed in natural populations. For example, in ISC *L*. *donovani* clinical isolates, the contrast was striking between the high genetic homogeneity (only 2,418 SNPs across the whole genome of 191 isolates) and the strain-specific aneuploidy, with several chromosomes showing a somy >2 [9]. Surprisingly, no correlation was found between somy and DR patterns in this study. This may be because these genomic studies were performed on promastigote forms, cultivated in vitro subsequent to their collection from patients, rendering it possible that the observed aneuploidy was selected during this postcollection cultivation.

This was supported experimentally in a recent study that followed chromosome number of *L*. *donovani* throughout the life cycle by sequencing different life stages, including amastigotes obtained from infected hamsters [81]. Starting from highly aneuploid (8 chromosomes with somy >2) in vitro promastigotes, all chromosomes but one showed a decrease in somy upon adaptation to growth in the hamster, while two new trisomies appeared, affecting chromosomes bearing genes essential for virulence, e.g., amastins and GP63 [81].

In brief, these data suggest that aneuploidy is adaptive [82] and sensitive to environmental conditions. Accordingly, chromosome amplification related to DR in patients could be diluted and disappear upon in vitro isolation and cultivation. This finding means that future WGS studies aiming to establish a link between genome features and phenotypes like DR should be performed on amastigotes directly isolated from clinical isolates rather than cultivating strains as promastigotes prior to analysis. This presents significant technical challenges given that parasite DNA derived directly from host is relatively scarce and at much lower levels than host DNA. However, advances in sequencing technology, including capturing *Leishmania* DNA before sequencing—e.g., with SureSelect technology—may offer a solution. Further discoveries on DR should, therefore, be expected from the application of this technology.

## Molecular mechanisms of DR to MIL

MIL is the first and only oral drug available against leishmaniasis. Since its registration in India in 2002, it has replaced the use of SSG as first-line treatment in the ISC [83,84], with cure rates higher than 94%. MIL, a phosphorylcholine ester of hexadecanol, was originally developed as an anticancer drug. The exact mode of action of MIL is not well understood, although it has been described to have a direct effect on the parasites by interfering with biosynthesis of phospholipids and metabolism of alkyl-lipids [85, 86], affecting mitochondrial cytochrome c oxidases and inducing mitochondrial depolarization and decrease of intracellular levels of ATP [87], and an apoptosis-like cell death [88–90].

The uptake of MIL and other alkyl-glycerophospholipids in *Leishmania* requires a translocation machinery that includes a P-type ATPase named the *Leishmania* miltefosine transporter (LMT), which is responsible for the translocation of phospholipids from the exoplasmic to the cytoplasmic leaflet of the plasma membrane of *Leishmania* [91]. The function of LMT depends on its binding to a specific B subunit of LMT called LRos3 [92], which belongs to the CDC50/LEM3 protein family. Both proteins are mutually dependent for their function and their localization at the plasma membrane of *Leishmania* [92, 93], being required for MIL uptake and susceptibility.

MIL has a long elimination half-life (approximately 120 h) that leads to subtherapeutic levels remaining for some weeks after a standard treatment course [94]. Following this observation, it was predicted that resistance to MIL would rapidly emerge in the regions where it was extensively used.

Ten years after the implementation of MIL in the ISC, its efficacy was shown to be decreasing with a relapse rate of 10% in India [7] and up to 20% in Nepal upon 12-month follow-up [8]. However, this increasing TF was not initially associated to drug resistance, with other factors invoked to explain the situation, including parasite virulence [95] and host factors [96]. Recently, however, two clinical isolates resistant to MIL were isolated in the ISC [10]. Although slower to emerge in the field than some had feared, laboratory-based experimentation has demonstrated that in vitro selection of promastigotes resistant to MIL was easily achieved [97–102].

The main mechanism of experimental resistance observed is associated with a significant reduction in drug internalization due, mainly, to a reduced uptake or an increased efflux of MIL. The acquisition of inactivating mutations or deletions in MIL translocation machinery LMT and/or LRos3 in *L*. *donovani* was shown to drastically increase MIL resistance in both in vitro and in vivo assays [91, 92, 103]. LMT and/or LRos3 have also been shown to represent MIL-resistant markers in clinical samples obtained from leishmaniasis patients showing therapeutic failure to MIL [10, 11,102].

In addition, the overexpression of ABC transporters ABCB4(MDR1), ABCG4, and ABCG6 has also been described to be associated with an increased resistance to several alkyl-lysophospholipids analogues, including MIL in *Leishmania* [104–106], due to a reduced intracellular accumulation because of increased efflux of the drug across the plasma membrane.

Other cellular modifications have also been proposed to contribute to MIL resistance in *Leishmania*. These include changes in the length and levels of unsaturation of fatty acids, as well as a reduction in ergosterol levels [107]; altered expression of genes involved in thiol metabolism, protein translation and folding, as well as DNA repair and replication machinery [93]; and higher ability to resist reactive oxygen species (ROS) [108] as well as a better tolerance towards, or reduced production of, oxidative stress [109, 110]. An increase in metacyclogenesis and infectivity has also been described in MIL-resistant promastigotes [109].

Recently, the use of omics techniques (whole-genome and RNA sequencing) in MIL experimental resistant *L*. *donovani* lines has revealed mutations in genes encoding proteins other than LMT. These include pyridoxal kinase and α-adaptin line protein as well as up- and down-regulation of specific genes associated with stress, membrane composition, and amino acid and folate metabolism [98, 100]. Specific roles in the drug’s mode of action or resistance mechanisms are not known, but a picture of a multifactorial process contributing to resistance to MIL is emerging.

## The molecular basis of AmB resistance in *Leishmania*

AmB has been used as an antifungal agent for the last 70 years [111] and as an anti-leishmanial since the 1960s [112]. Its ability to bind to ergosterol-related sterols in cell membranes explains its specificity [113]. Because *Leishmania* parasites, in common with fungi, use ergosterol as a primary membrane sterol, they too are sensitive to this drug. Mammalian cells use cholesterol instead and are accordingly less sensitive to the drug. AmB is a natural product produced by *Streptomyces nodosus* and has an amphipathic nature with hydrophilic and hydrophobic moieties [113]. Once within the vicinity of the membrane, it spontaneously assembles with its hydrophobic surface in contact with membrane lipids while hydrophilic surfaces of adjacent molecules produce a pore. The exchange of ions across the surface via the pores contributes to cell death [114]. However, the drug also induces oxidative stress, and binding to sterol per se irrespective of pore formation also contributes to the death of yeast cells [115, 116].

WHO has been promoting the use of single-dose Ambisome, a relatively harmless liposomal formulation of the drug for VL patients, particularly on the ISC. This campaign, which enables the widespread use of the drug without needing patient hospitalization and repeated injections, has clear advantages from a public health perspective [117–119]. However, the dose available in this single shot is not far from the minimum required to treat the disease [118]. This poses the risk that this single shot, not being always curative, could select for parasites with reduced vulnerability to the drug. These may then transmit as a less sensitive population, itself then potentially capable of developing further resistance. As discussed below in the section on combination therapies, AmB is currently recommended as a potential partner drug in a number of regimens. If resistance genes to AmB are selected during a monotherapy phase, there is the risk that they will render ineffective the AmB part of any combination. In that case, the partner drug will be effectively used as monotherapy and—worse still—possibly be used against parasites for which that drug is used at doses that are suboptimal for monotherapy, while assuming that the AmB part is effective (i.e., in combination therapies, drugs are often given at lower doses than in monotherapy). Thus selection of resistance to the second drug as well becomes increasingly likely.

Resistance, however, has been considered of low risk for AmB. This is partly because resistance has been relatively rare in fungal infections, in spite of 70 years of use [111]. Moreover, reports of AmB resistance in leishmaniasis have also been rare.

Notwithstanding, there are multiple reports of AmB resistance in fungal infections [e.g., 119–123]. Moreover, the first cases of TF with AmB have already appeared in India [124, 125], where resistant parasites were clearly associated with one case [124]. In France, TF in HIV–*Leishmania* coinfections has been reported [126], and AmB unresponsiveness in an immunosuppressed patient in Switzerland was reported as well [127]. In laboratory studies, it has been possible to select for resistance to AmB in several species of *Leishmania*, and both promastigote and amastigote forms of the parasite resistant to AmB have been selected [124, 128, 129]. It seems, therefore, that serious attention should be given to the risk of selecting resistance to AmB in *Leishmania*.

Several studies have started to elucidate the mode of action and resistance mechanisms to the drug. For example, it was shown that treatment with AmB permeabilized leishmanial lipid bilayers to ion and dye exchanges [114, 130], indicating that the drug binds to the membrane as in yeast. A number of selected resistant lines revealed changes in the sterol content. Notably, ergosterol and related sterols were replaced by cholesta-related sterols. In one case, this was attributed to possible changes in sterol methyltransferase, causing disruption in the sterol pathway and accumulation of intermediates in the ergosterol synthetic pathway [124, 131]. Several other studies have shown that loss of ergosterol is associated with resistance [128, 129], which links to the drug’s dependency on binding this sterol to exert its mode of action. It has recently been demonstrated that mutations to the gene encoding sterol 14α-demethylase underlie resistance [132] with an accumulation of that enzyme’s product, which indicates that the demethylase is still active itself, but its product no longer enters the remainder of the pathway.

Other studies have also indicated separate changes associated with resistance. For example, a number of selected lines have increased parts of their oxidative defense mechanism [128, 133, 134], which indicates that part of the drug’s mode of action is via the induction of oxidative stress and that prevention of this oxidative damage can yield a reduced sensitivity to the drug. In another resistant line, alterations to the MIL transporter were identified, and in this case, cross-resistance between AmB and MIL was detected, although the functional impacts of these mutations were unclear because the impact on MIL uptake was minimal [135]. This may be attributed to changes in the lipid composition of the membrane because the MIL transporter plays a key role in arranging lipids within the membrane; indeed, extensive changes to lipid profile were observed in this resistant line and were partially restored by complementation with wild-type MIL transporter [135].

It appears, therefore, that resistance to AmB can be selected in *Leishmania*. Although it appears that this is more readily achieved in promastigote forms than in amastigote forms (possibly because a combination of changes in membrane sterol composition and response to oxidative stress are required), the latter form can indeed develop resistance to the drug, and this has been found already in cases of TF. More work is required to collect a comprehensive inventory of the mechanisms that can underpin resistance and to help develop a tool kit that can assist in diagnosing potentially resistant lines in the field.

## Resistance to drug combination therapy in *Leishmania*

In most antimicrobial drug scenarios, there is a growing awareness that combination therapies offer substantial benefits, including overcoming resistance. Using at least two drugs with different mechanisms of action should also improve the control of leishmaniasis. In the case of malaria, AIDS, and tuberculosis, substantial effort has gone into seeking optimal combination therapy based on both mode of action of partner drugs and their pharmacokinetic parameters. Characteristics such as parasite clearance time and drug half-life are guiding the rational selection of combinations. So far, relatively little has been done with respect to combination therapy in *Leishmania*, although preferred pairings are emerging [136–139].

WHO recommendations [140] for combination therapy in Indian VL patients are for a single dose of liposomal AmB together with MIL [136, 141] or a single dose of liposomal AmB plus paromomycin [136]. SSG and paromomycin together comprise the WHO recommendation for Sudan [137, 142]. Finally, for other East African countries, WHO recommends several combination therapies, including a single dose of liposomal AmB followed by MIL [143], or AmB administered simultaneously with SSG [138], or SSG plus paromomycin [142].

The efficacy of combination therapy must be established for each *Leishmania* species, and it is increasingly appreciated that it should be implemented for all clinical manifestations of leishmaniasis, including CL, in order to obtain lower relapse rates in comparison with the use of monotherapy.

Resistance to individual drugs in *Leishmania* appears to arise easily, partly due to plasticity in their genome [69, 73]. Whether drug combinations can also select resistant *Leishmania* has also been explored. At least under our experimental conditions, *L*. *donovani* promastigotes were shown to develop resistance even to combinations [12], particularly to MIL/paromomycin and SSG/paromomycin pairings [12]. These results were confirmed in intracellular amastigotes [12] although intrinsic technical difficulties—including low replication rates and the limited time of survival of infected cell cultures—limit the time available to select resistant lines.

Metabolomics analysis was used to investigate *Leishmania* lines resistant to different drug combinations [144] alongside the investigation of the phenotypic adaptations and fitness of parasites resistant to drug combinations [145]. These studies suggest that combination-resistant lines develop metabolic changes in multiple pathways, including proline and lipid metabolism [144], thereby activating stress responses—including enhanced ability to neutralize drug-induced ROS production and decreases in membrane fluidity [144]. Drug-combination–resistant parasites are more tolerant to ATP loss, have increased thiol levels, resist depolarization of the mitochondrial membrane, show no DNA fragmentation under drug pressure, and sustain membrane integrity.

Using a transgenic *L*. *donovani* line expressing luciferase (LUC) as a reporter to assess viability and dynamics of mixed populations of wild-type and combination-therapy–resistant parasites, it was observed that combination-resistant parasites acquire an overall increase in fitness over wild type under different stress situations, including nutrient starvation and heat-shock pH stress and thus have the ability to survive as intracellular amastigotes [145].

In conclusion, these results clearly suggest that, although it is more difficult for *Leishmania* to acquire resistance to combination therapy over monotherapy, resistance to combinations is possible (especially when paromomycin is one of the partner drugs). This has important clinical relevance for the use of combination therapies and their impact on leishmaniasis control programs.

## General conclusions

SSG TF offers a challenge to our efforts to control the leishmaniases. Several factors have contributed to diminished efficacy of drugs registered for use against these diseases, including changes in host immunity associated with the global HIV/AIDS epidemic and a changing demography in the range of the disease. Molecular mechanisms for resistance have all been determined in laboratory-selected lines, and the advent of new technologies, particularly next-generation sequencing, is increasing our ability to understand resistance in the field. In the case of SSG resistance in India, a clear pattern of genetic change associated with resistance is known. The discovery of genes underpinning resistance to other drugs, including MIL and AmB, will similarly offer an ability to monitor the emergence and spread of resistance to these drugs in the field as well. This will inform surveillance strategies to monitor drug efficacy and should feed into coordinated measures to enable further research and the setting of policies to optimize our capability to continue efforts to control the leishmaniases as a global health problem [146].

**Key learning points**

1. TF goes far beyond DR, therefore these terms are not synonyms. Additionally, host and parasite factors conspire to affect outcomes of treated *Leishmania* infections.
2. WGS provides enough resolution to help clarify the molecular epidemiology of *L*. *donovani* in the Indian subcontinent and in the context of DR. This experience might influence how information is gathered on emergence and spread of resistance to other drugs and reinforce the need to develop leishmaniasis surveillance and control programs.
3. The use of combination therapies is relevant for leishmaniasis control programs at the clinical level because it is more difficult for *Leishmania* to acquire resistance to combination therapies than to monotherapy. However, resistance to combinations is easy to develop, especially if paromomycin is one of the partner drugs.
4. Although AmB is currently recommended as a potential partner drug in a number of regimens, if resistance genes to AmB are selected during a monotherapy phase, there is a risk that the AmB part of any combination will be rendered ineffective.
5. The lessons drawn from the molecular epidemiology analysis of the case of SSG resistance in leishmaniasis might be helpful for defining appropriate use of the few additional available drugs and in the design and implementation of adequate surveillance strategies at the level of primary health centers, reference hospitals, and laboratories.

**Top five papers**

1. Sundar S, Sinha PK, Rai M, Verma DK, Nawin K, Alam S, et al. Comparison of short-course multidrug treatment with standard therapy for visceral leishmaniasis in India: an open-label, non-inferiority, randomised controlled trial. Lancet. 2011;377(9764):477–86. doi: 10.1016/S0140-6736(10)62050-8
2. Lopez-Velez R, Perez-Molina JA, Guerrero A, Baquero F, Villarrubia J, Escribano L, et al. Clinicoepidemiologic characteristics, prognostic factors, and survival analysis of patients coinfected with human immunodeficiency virus and *Leishmania* in an area of Madrid, Spain. Am J Trop Med Hyg. 1998;58(4):436–43
3. Purkait B, Kumar A, Nandi N, Sardar AH, Das S, Kumar S, et al. Mechanism of amphotericin B resistance in clinical isolates of *Leishmania donovani*. Antimicrob Agents Chemother. 2012;56(2):1031–41. doi: 10.1128/AAC.00030-11
4. García-Hernández R, Manzano JI, Castanys S, Gamarro F. *Leishmania donovani* develops resistance to drug combinations. PLoS Negl Trop Dis. 2012;6(12):e1974. doi: 10.1371/journal.pntd.0001974
5. Imamura H, Downing T, Van den Broeck F, Sanders MJ, Rijal S, Sundar S, et al. Evolutionary genomics of epidemic visceral leishmaniasis in the Indian subcontinent. Elife. 2016;5. pii: e12613. doi: 10.7554/eLife.12613
